# Exposure to cooking emissions in kitchens and chronic obstructive pulmonary disease in women: a population-based case-control study in Ouagadougou, Burkina Faso

**DOI:** 10.11604/pamj.2024.49.88.43238

**Published:** 2024-11-22

**Authors:** Adama Sana, Benoit Kafando, Nicolas Meda, Catherine Bouland

**Affiliations:** 1 Département Biomédical et Santé Publique, Institut de Recherche en Sciences de la Santé (CNRST/IRSS), Ouagadougou, Burkina Faso,; 2 Département de Santé Publique, Université Joseph Ki-Zerbo, Ouagadougou, Burkina Faso,; 3 Centre de Recherche en Santé Environnementale et Santé au Travail, Ecole de Santé Publique, Université Libre de Bruxelles, Brusselles, Belgique

**Keywords:** Chronic obstructive, pulmonary disease, environmental pollutants, particulate matter, female

## Abstract

**Introduction:**

women exposed to smoke are three times more likely to suffer from chronic obstructive pulmonary disease than women who cook with clean fuels. The present study aims to compare the level of exposure to particulate matter (PM_2.5_), carbon monoxide (CO), and total volatile organic compounds (VOCs) inside kitchens, between women with chronic obstructive pulmonary disease (COPD) and healthy women, in Ouagadougou, Burkina Faso.

**Methods:**

a pilot case-control study was conducted from January 7^th^ to 25^th^, 2020, in 2 neighborhoods of Ouagadougou. Cases were women diagnosed with COPD and controls were women without COPD. The diagnosis of COPD was made on the basis of a standardized questionnaire administered and followed by a spirometry test. The sample comprised 9 cases and 9 controls. Exposure was assessed by using a multi-pollutant hand-held device, over a 24-hour period. Exposures of cases and controls were compared using the independent Student's t-test and in cases where the distribution was not normal, the Kruskal-Wallis test was used.

**Results:**

the mean age was 59 ± 9.86 years in the cases group and 58.56 ± 7.45 years in the control group and there is no significant difference (p = 0.757). The mean concentrations of PM_2.5_ measured in the 2 groups were above the World Health Organization (WHO) recommended threshold of 15 µg/m^3^ for 24 h exposure. The mean PM_2.5_ concentration was 127.10 µg/m^3^ in the cases and 16.23 µg/m^3^ in the control group (p= 0.133). Concentrations of CO and VOCs were also higher in the kitchens of the cases than in those of the controls. However, no differences were statistically significant.

**Conclusion:**

although no statistically significant differences were observed, pollutant concentrations were higher in the kitchens of women with COPD. In addition, PM_2.5_ levels measured in both groups exceeded WHO-recommended thresholds, underlining the need to reduce household exposure to pollutants. Further research is needed to better understand these impacts.

## Introduction

In recent years, research has shown an increase in morbidity and mortality associated with non-communicable diseases (NCDs), particularly in most developing countries [1,2]. Chronic obstructive pulmonary disease (COPD) is one of the most common NCDs. In 2004, the World Health Organization (WHO) estimated that over 64 million people worldwide suffered from COPD [3]. With 3.23 million deaths in 2019, COPD is the third leading cause of death worldwide [3]. The overall prevalence of the disease is increasing faster in women than in men. Over the past two decades, COPD mortality rates have also risen more rapidly in women, and since 2000, more women than men have died from COPD [4]. COPD is a preventable and treatable public health problem.

Epidemiological studies have shown a strong association between the use of biomass as fuel and the onset of COPD in women [5-7]. Women exposed to smoke are three times more likely to suffer from COPD than women who cook with clean fuels such as liquefied petroleum gas (LPG), and electricity [8-11].

According to the WHO, a third of the world's population, or 2.6 billion people, still do not have access to clean cooking, persisting in the use of polluting fuels (wood, agricultural residues, animal dung, coal, charcoal) and inefficient technologies, which constitute a health hazard and a major cause of illness and death, particularly for women and children in low- and middle-income countries [3]. The smoke emitted is composed of hazardous substances such as particulate matter (PM_2.5_), carbon monoxide (CO), and polycyclic aromatic hydrocarbons (PAHs) [10,12]. In Burkina Faso, 65.6% of households use firewood with a traditional stove for cooking and they are 16.1% using gas or biogas, and 10.8% use charcoal [13]. In urban areas, 41.5% of households use gas or biogas for cooking, however, the majority of households still use biomass fuels, particularly firewood and charcoal, for preparing meals [13].

For better prevention of the disease, it is essential to identify the risk factors. The aim of the present study was to analyze the relationship between exposure to indoor pollutants and COPD in Ouagadougou, in Burkina Faso. The specific objectives included a measure of the concentration of PM_2.5_, CO, and volatile organic compounds (VOCs) (cooking-generated pollutants) in household kitchens. We have also compared the mean concentration between cases and control groups to assess the association between the exposure to PM_2.5_, CO, and VOCs in the kitchen and COPD.

## Methods

**Study design and setting:** a population-based case-control study was conducted from January 7^th^ to January 25^th^, 2020, with the aim of comparing the level of concentration of certain pollutants in the kitchen as an exposure variable between a group of women with COPD identified during the previous phase of the project in which the present study falls, and another group of control women free of COPD. The study took place in Sector 15 (Kilwin district) and Sector 17 (Tanghin district) located respectively in arrondissements 3 and 4 of the city of Ouagadougou, Burkina Faso's main city. Burkina Faso is a landlocked Sudano-Sahelian country situated in the heart of West Africa and is bordered by Mali to the northwest, Niger to the northeast, Benin to the southeast, Togo and Ghana to the south, and the Ivory Coast to the southwest. The population is estimated at 20,505,155 inhabitants in 2019, and women represent 51.7%. With an urbanization rate of 26.1%, the total population of cities in Burkina Faso was 5,360,112 in 2019 [13]. Ouagadougou, the country's capital, is the largest city with a population of 2,415,266 inhabitants. In Burkina Faso, 64.2% of the population is younger than 24 years, and 3.4% is aged 65 years and older [13]. Burkina Faso is divided into 13 regions which in turn are divided into 45 provinces. Ouagadougou is the capital city, located in "Centre" Region, and Bobo-Dioulasso, the country´s economic-city located in the "Hauts-Bassins" Region. Ouagadougou has 12 arrondissements and 55 sectors.

**Study population:** the study involved women aged at least 18 and in charge of cooking in their household. Cases were women in whom the diagnosis of COPD was confirmed after post-bronchodilation spirometry. Controls were selected from women with normal lung function confirmed by spirometry. Cases were matched to controls. In that respect, for each case, a control corresponding to the case in terms of age (+ or - 5 years) and area of residence was identified.

**Data collection:** the fuel used for cooking, and the socio-demographic, and personal characteristics of the woman participants were collected by a standardized questionnaire. Respiratory health data were also collected. Respiratory health information collected from the participant concerned the symptoms of obstructive respiratory diseases such as COPD including chronic bronchitis (CB), and asthma. Details of the techniques and tools used to diagnose COPD, BC, and asthma in our study are available in previously published articles [14,15]. Spirometry was performed by two final-year medical students trained by a technician performing spirometry tests in the university hospital Yalgado Ouedraogo (CHU/YO). The results were reviewed by a pulmonologist. Environmental data were collected and recorded on a specially designed grid (kitchen location, type of fuel used, cooking time, number of meals prepared on the day of measurement, pollutant concentrations). Pollutant concentrations were measured using a QUEST EVM 7 portable air pollutant measuring device. The equipment and method used for measuring pollutant concentration was detailed in a previous article [16]. It is fitted with a laser photometer that measures dust concentration over time by reflection of the light beam. It simultaneously measures concentrations of volatile organic compounds (VOCs), CO, carbon dioxide (CO_2_), relative humidity, temperature and dew point. Instantaneous values are measured every second. For each parameter measured, it calculates minimum, average, and maximum values. During measurements, the device is mounted on a metal support at a height of 1.5 meters from the ground and at a distance of 1.5 meters from the fireplace, for 24 hours. The conditions under which the device was installed are in line with those used by other authors [12,16-18]. The device is assembled, installed, and recovered from participants´ homes by two trained technicians. To estimate cooking time, we asked the person in charge of cooking the meals to note down, or ask someone who knows how to do so, the start and end times. In addition to these variables, meteorological data such as temperature and humidity were also collected. The instrument was calibrated prior to the measurement period.

**Definitions:** variables included the study population (women in charge of the cooking in the household) characteristics (age, education, occupation, place of residence, weight, and height), household characteristics (number of people in a household, kitchen location, mean cooking duration, cooking fuel type, frequency of meal preparation per day), environmental data (PM_2.5_, CO_2_, CO, COV, temperature, relative humidity, dew point), respiratory health variables that are the outcome variables (respiratory symptoms, respiratory function: Forced Expiratory Volume at one Second (FEV1), Forced Vital Capacity (FVC), Peak Expiratory Flow (PEF), FEV1/FVC ratio). COPD was defined as a post-bronchodilation FEV1/FVC (forced expiratory volume in 1 sec/forced vital capacity) ratio less than 0.70.

**Statistical analysis:** data were entered using EpiData software. The software Stata/SE version 12.0 was used for data analysis. Descriptive and explanatory analyses were carried out. The descriptive analysis was used to present sociodemographic characteristics, and meal preparation conditions. For quantitative variables such as age, and cooking duration, we calculate mean and standard deviation in cases and control groups. For spirometry values, we also calculate the mean and range in the two groups. For analytical statistics aimed to measure the association between 24-hour average emissions in kitchens and COPD, cases and controls were compared using the independent Student's t-test, which allows comparison of the mean of 2 groups of independent samples when the distribution is normal, and in cases where the distribution is not normal the Kruskal Wallis test was used. For the comparison of categorical/categorical variables between the 2 groups, we used the chi-square test or the Fisher exact test. Normality was verified using histograms. A p-value less than 0.05 is considered to be statistically significant.

**Ethical consideration:** a signed informed consent was requested to all participants, also for the heads of households in cases where the latter was not the participant. In some cases, consent was translated and explained in the language of the participant. The project received the approval of the Burkina Faso Ethics Committee for Health Research, deliberation number: 2015-9-114.

## Results

**Participants:** a total of 564 women performed and completed a valid spirometry and twelve cases of COPD were diagnosed. These 12 cases were matched with 12 controls. However, of the 12 cases, 1 was traveling during the pollution exposure measurement phase and 2 could not be reached. Finally, 9 cases and 9 controls were included in the study. Exposure information was collected on all these 9 cases and theirs matched 9 controls.

**Descriptive analysis:** the mean age was 59 ± 9.86 years in cases group and 58.56 ± 7.45 years in controls group. Biomass fuel was exclusively used in 7 of the 9 cases (77.78%) and by 3 of the controls (33.33%). Average cooking time was 303.56 ± 123.34 minutes in cases and 356.78 ± 163.79 minutes in controls groups. All cases cook at most twice a day. The characteristics of the study population and spirometry values in the case and control groups are shown in Table 1 and Table 2 respectively.

**Table 1:** characteristics of the case and control populations

Variables	Cases (n=9)	Controls (n=9)	P-value
Mean age in years (SD)	59 ± 9.86	58.56 ± 7.45	0.757
**Place of residence**			**1.000**
Kilwin	5 (55.56 %)	5 (55.56 %)	
Tanghin	4 (44.44 %)	4 (44.44 %)	
**Fuel**			**0.214**
Gas	1 (11.11 %)	2 (22.22 %)	
Biomass	7 (77.78 %)	3 (33.33 %)	
Biomass and gas	1 (11.11 %)	4 (44.44 %)	
**Kitchen**			**0.613**
Free air	5 (55.56 %)	4 (44.44 %)	
External	3 (33.33 %)	3 (33.33 %)	
Internal	1 (11.11 %)	2 (22.22 %)	
Mean cooking time (minutes)	303.56 ± 123.34	356.78 ± 163.79	0.426
**Frequency of meal preparation per day**			**0.576**
1 time	2 (22.22 %)	1 (11.11 %)	
2 times	7 (77.78 %)	6 (66.67 %)	
3 times	0 (00.00 %)	2 (22.22 %)	

SD: standard deviation

**Table 2:** spirometric values (without bronchodilator administration) in cases and controls

Variables	Cases (n=9)		Controls (n=9)	
	Mean (SD)	Min-max	Mean (SD)	Min-max
FEV1 (liter)	1.456 (0.615)	0.63-2.79	2.32 (0.359)	1.61-3.02
% predicted FEV1	2.572 (0.371)	2.02-2.95	2.658 (0.344)	1.96-3.14
FVC (liter)	2.589 (0.798)	1.86-4.37	2.905 (0.537)	2.19-3.82
% predicted FVC	3.28 (0.463)	2.52-3.74	3.417 (0.400)	2.57-4
PEF (liter)	2.869 (1.126)	1.20-4.36	4.913 (1.181)	3.7-7.39
% predicted PEF	6.298 (0.653)	5.27-6.97	6.436 (0.593)	5.27-7.28
FEV1/ FVC	55.511(11.537)	27.4-66.2	80.522 (8.145)	67.4-92.5
% predicted FEV1/ FVC	79.122 (2.298)	76.1-83.0	78.365 (1.583)	76.57-81.25

FEV1: forced expiratory volume at one second; FVC: forced vital capacity; PEF: peak expiratory flow; SD: standard deviation; %: percentage

The mean PM_2.5_ concentration was 127.10 (256.20) µg/m^3^ in the case group and 16.23 (46.43) µg/m^3^ in the control group. The minimum level of PM_2.5_ measured was 91.27 (241.84) µg/m^3^ and the maximum was 1572.41 (3646.74) µg/m^3^ in the cases group. However, the minimum level of PM_2.5_ measured in the control group was 2.22 (6.29) µg/m^3^ and the maximum was 509.77 (1433.56) µg/m^3^. The mean concentrations of particulate matter less than 2.5 µg measured in the 2 groups were above the WHO recommended limit value of 15 µg/m^3^.

The average CO concentration was 18.33 (27.99) ppm in the case group and 5.89 (14.80) ppm in the control group. The average concentrations of CO in the 2 groups were above the WHO recommended limit value of 4 mg/m^3^ (corresponding to 3.49 ppm). The average VOCs concentration was 47.46 (84.55) ppm in the case group and 17.36 (52.07) ppm in the control group.

**Bivariate analysis:** it revealed that there were no statistically significant differences in mean concentrations of PM_2.5_ (p = 0.133), CO (p = 0.309), and VOCs (p = 0.377), between the cases and the controls groups (Table 3). Also, differences of minimum and maximum concentrations of PM_2.5_, CO, and VOCs in case kitchens and control kitchens were not statistically significant in the bivariate analysis (Table 3).

**Table 3:** bivariate analysis (comparison of mean exposure levels between cases and controls)

Variables	Cases	Controls	P-value	WHO Rec.
	Mean (SD)	Mean (SD)		
**PM2.5 (µg/m^3^)**				
Mean	127.10 (256.20)	16.23 (46.43)	0.133	15 µg/m^3^
Minimum	91.27 (241.84)	2.22 (6.29)	0.627	
Maximum	1572.41 (3646.74)	509.77 (1433.56)	0.122	
**CO (ppm)**				
Mean	18.33 (27.99)	5.89 (14.80)	0.309	4 mg/m^3^
Minimum	5.22 (10.02)	1.33 (4)	0.303	
Maximum	67.11 (76.66)	43 (53.01)	0.479	
**VOC (ppm)**				
Mean	47.46 (84.55)	17.36 (52.07)	0.377	
Minimum	27.74 (45.57)	9.29 (27.87)	0.315	
Maximum	74.92 (129.39)	29.95 (82.73)	0.393	
**T° and RH**				
Mean Tº (ºC)	28.47 (3.84)	29.74 (1.74)	0.377	
Mean RH (%)	29.56 (3.74)	29.31 (5.11)	0.909	

SD: standard deviation; PM: particulate matter; CO: carbon monoxide; VOC: total volatile organic compounds; T°: temperature; RH: relative humidity; ppm: parts per million; WHO Rec.: World Health Organization recommandation

**Multivariate analysis:** the results obtained in the bivariate analysis do not allow us to perform a multivariate analysis.

## Discussion

This pilot study assessed and compared levels of exposure to fine particulate matter, carbon monoxide, and volatile organic compounds between women with chronic obstructive pulmonary disease and healthy women in the city of Ouagadougou, Burkina Faso. The average PM_2.5_ concentration was 127.10 μg/m^3^ in the case group and 16.23 μg/m^3^ in the control group. Concentrations of CO and VOCs were also higher in the kitchens of the cases compared to the controls. However, the differences observed were not statistically significant. Furthermore, PM_2.5_ levels in both groups exceeded the thresholds recommended by the World Health Organization.

In their study of Taiwanese non-smokers, Huang *et al*. observed that exposure to PM_2.5_ at concentrations greater than 38.98 μg/m^3^ increased susceptibility to COPD [19]. According to WHO guidelines on indoor air quality [20], the recommended limit value for PM_2.5_ is 15 μg/m^3^(24-hour average). The average concentrations of particulate matter less than 2.5 μg measured in the 2 groups were above the WHO recommended limit value of 15 μg/m^3^. Also, while the mean PM_2.5_ concentration in the controls was slightly above the WHO guideline value, that in the cases was 8 times higher than the WHO recommended value. In fact, almost 77% of cases used only biomass fuels (wood and charcoal) for cooking, compared with 33% of controls. However, 44% of controls used a combination of biomass and gas for cooking. Average PM_2.5_ concentrations measured in households were 78.88 μg/m^3^, 64.86 μg/m^3^ and 61.33 μg/m^3^ in households using biomass fuel, gas (LPG) and the biomass/gas combination respectively.

These high concentrations in kitchens where biomass is the fuel used have been confirmed by other studies. In India, Mukherjee *et al*. found mean PM_2.5_ concentrations of 298 ± 83 μg/m^3^ and 82 ± 28 μg/m^3^ in biomass-fueled kitchens during cooking and non-cooking hours respectively, compared with concentrations of 79 ± 22 μg/m^3^ and 42 ± 12 μg/m^3^ in households using LPG for cooking [21]. In China, Hu *et al*. found that the burning of wood and other plant matter such as tobacco stalks and corn cobs was responsible for high personal exposure to PM_2.5_ of the order of 289 and 225 μg/m^3^ in Xuanwei and Fuyuan respectively [22]. Few studies objectively measuring exposure to indoor household pollutants related to cooking fuels have been conducted in Africa. Among the African studies, one conducted in Nigeria found a mean PM_2.5_ concentration of 1575.1 μg/m^3^ (interquartile range (IQR) 943.6-2847.0) in households using wood exclusively as cooking fuel [23].

In addition, studies have shown different concentrations depending on the type of biomass fuel used. Taylor *et al*. [24], in their study conducted in Sierra Leone in 2011, found that mean concentrations of suspended microparticles were significantly higher in kitchens of dwellings where wood was used, compared with kitchens where charcoal was burnt: mean (SD) = 882.4 (518.0) versus 197.2 (136.0) μg/m^3^, with a p-value of 0.003. Ingale *et al*. in India showed that the mean PM_10_ concentration is higher in households using agricultural residues compared with those using wood: 4423 +/- 2793 versus. 6285 +/- 3996 μg/m^3^ [25]. Research in Uganda and Ethiopia has shown that PM_2.5_ concentrations are highest when animal dung is burnt, followed by agricultural residues and then wood [26]. Ibhafidon *et al*. in Nigeria found that LPG was the least polluting fuel (particularly in terms of micro-particle emissions) compared with kerosene and firewood [27].

During combustion (pyrolysis), wood breaks down into gases (CO, H_2_, CH4), liquids (tars), and solids (coal). The gases and some of the charcoal then undergo combustion. Tars, which contain mainly aromatic hydrocarbons (PAHs), do not burn directly, but are released with the smoke [28]. Unburned coal microfragments form fine particles classified into three categories: PM_1_< 1 micron, PM_2.5_ < 2.5 microns, and PM_10_ < 10 microns [28]. Emissions of particulates, CO, NMVOCs, and polycyclic aromatic hydrocarbons (PAHs) are highly dependent on the efficiency of the stoves used, the amount of air present during combustion, and the moisture content of the wood. Emissions of these pollutants decrease as the performance of wood-burning equipment increases [29,30].

Open fireplaces have very low energy efficiency, generally between 10 and 20% in the form of useful heat [28]. Charcoal is much more energy-efficient than wood, for the same quantities. The calorific value of wood averages 3500 Kcal/kg of wood and around 7500 Kcal/kg for charcoal, while the calorific value of petroleum products averages 10000 Kcal/liter of fuel [31].

Pollutant concentrations can be amplified by site topography, including the location of the kitchen, and by meteorological conditions such as the presence of an inversion layer preventing vertical dispersion of the various pollutants. In our study, concentrations were higher in separate (external) kitchens, where the average PM_2.5_ was 163.52 μg/m^3^, compared with an average of 55.46 μg/m^3^ in internal kitchens and 15.83 μg/m^3^ in open-air kitchens. This result runs counter to what other studies have found. Indeed, Kafando *et al*. found that PM_2.5_ concentrations were significantly higher (bivariate and multivariate analyses) in households where cooking took place in the open air [16]. Our results can be justified by the fact that, when cooking is done indoors (enclosed internal or external kitchen), in the absence of an effective ventilation system, the smoke released can remain suspended in the internal atmosphere for long periods. Also, in the population studied, while gas is the fuel used in internal kitchens and wood is often used for open-air cooking, in external kitchens, which are often poorly ventilated, both biomass fuel and butane gas are burned. The type of cooking can increase indoor particulate concentrations by more than five times [32]. In addition, PM_2.5_ concentrations could be increased by up to 3, 30, and 90 times respectively of background levels during smoking, frying, and grilling, respectively [33].

We found a difference in PM_2.5_ concentration between COPD cases and controls. However, there was no significant association between PM_2.5_ concentration and COPD. Nevertheless, several studies have reported that exposure to particulate matter is significantly associated with reduced lung function [34-36] and risk of COPD-like symptoms [37,38], chronic bronchitis and COPD [39,40].

The major limitation of the present study was the low sample size, which may reduce the ability to detect a significant difference between cases and controls. Thus, the observed results from the bivariate analysis can be explained by the reduced statistical power induced by our small sample size. In fact, as a reminder, only 12 cases of COPD had been diagnosed, and it had been possible to measure pollutant concentrations in only 9 cases and the 9 controls associated with them. Studies involving larger numbers of cases and controls are needed. Also, these are 24-hour averages which are probably not representative of the concentrations emitted during meal preparation hours and the associated exposures.

## Conclusion

The average PM_2.5_ concentration was higher in the kitchens of the case group compared to the control group, although the difference was not statistically significant. PM_2.5_ levels in both groups exceeded the thresholds recommended by the WHO, with the case group recording concentrations more than eight times higher than the WHO guideline. Biomass fuel was used exclusively by 77.78% of the case group and 33.33% of the control group. These findings highlight the critical need to transition to cleaner fuels to reduce household pollutant exposure and lower the risk of COPD among women responsible for cooking. However, due to the limited sample size, caution is advised when interpreting these results. Further studies with larger sample sizes and repeated measurements over an extended period are necessary to confirm these findings and better understand the relationship between pollutant exposure and COPD development.

### What is known about this topic

- Chronic obstructive pulmonary disease is one of the most common NCDs;
- Chronic obstructive pulmonary disease is the third leading cause of death worldwide;
- Some studies have shown a strong association between the use of biomass as fuel and the onset of COPD in women.

### What this study adds

- This study provides data on the level of exposure to PM_2.5_, CO, and VOCs in kitchens in the African context where air quality data are scarce;
- This study assessed the relationship between exposure to PM_2.5_, CO and VOCs and COPD in women;
- The mean concentration of PM_2.5_ measured in the study population was above the World Health Organization (WHO) recommended threshold of 15 µg/m^3^ for 24h exposure.
